# An Integrated Evaluation Framework for Adult Heart Regeneration

**DOI:** 10.1111/jcmm.71099

**Published:** 2026-03-23

**Authors:** Yixun Li, Xinchang Liu, Xianliang Zhou, Yu Nie, Jie Feng

**Affiliations:** ^1^ State Key Laboratory of Cardiovascular Disease, Fuwai Hospital, National Center for Cardiovascular Disease Chinese Academy of Medical Sciences and Peking Union Medical College Beijing China; ^2^ National Health Commission Key Laboratory of Cardiovascular Regenerative Medicine, Central China Subcenter of National Center for Cardiovascular Disease, Henan Cardiovascular Disease Center, Fuwai Center‐China Cardiovascular Hospital Central China Fuwai Hospital of Zhengzhou University Zhengzhou China; ^3^ Shenzhen Key Laboratory of Cardiovascular Disease, Fuwai Hospital Chinese Academy of Medical Sciences Shenzhen China

## Abstract

Myocardial infarction (MI) remains a primary global cause of mortality due to irreversible cardiomyocyte loss. While promoting endogenous regeneration offers a highly promising therapy, its validation is critically hindered by the lack of standardised, comprehensive methodologies for quantitative assessment. To address this, we developed an integrated, systematically validated experimental framework for the precise evaluation of heart regeneration after ischemic injury in adult mice. This framework standardises four key assessment dimensions: (1) serial echocardiography mapping for precise serial evaluation of left ventricular function and structural changes; (2) standardised Masson's trichrome staining with systematic transverse sampling across five sections for reproducible quantification of post‐ischemic scar burden; (3) stringent multi‐marker strategy (EdU, pH 3 and Aurora B) for the accurate quantification of cardiomyocyte cell‐cycle reactivation; (4) total cardiomyocyte enumeration achieved by employing enzymatic digestion to derive definitive quantitative metrics, confirming successful cell replenishment. This integrated framework establishes detailed and reproducible methodological standards to significantly enhance the accuracy and reliability of heart regeneration research. It thereby provides a robust reference for the standardised assessment of heart regeneration in adult mice.

**Abbreviations**
bpm: beats per minute
CPC: chromosomal passenger complex
cTnT: Cardiac Troponin T
EdU: 5‐ethynyl‐2′‐deoxyuridine
EF: ejection fraction
FS: fractional shortening
HR: heart rate
I/R: ischemia/reperfusion
IVS; d: interventricular septum thickness at end‐diastole
IVS; s: interventricular septum thickness at end‐systole
LV: left ventricular
LVAD: left ventricular assist device
LVAW; d: left ventricular anterior wall thickness at end‐diastole
LVAW; s: left ventricular anterior wall thickness at end‐systole
LVID; d: left ventricular internal diameter at end‐diastole
LVID; s: left ventricular internal diameter at end‐systole
LVPW; d: left ventricular posterior wall thickness at end‐diastole
LVPW; s: left ventricular posterior wall thickness at end‐systole
MADM: mosaic analysis with double markers
MI: myocardial infarction
PBS: phosphate‐buffered saline
PFA: paraformaldehyde
pH3: phospho‐histone H3
PLAX: parasternal long‐axis
PSAX: parasternal short‐axis

## 1 Introduction

Ischemic heart disease, particularly myocardial infarction (MI), remains a leading global cause of mortality due to irreversible cardiomyocyte loss and subsequent pathological remodelling [1]. Promoting cardiomyocyte proliferation to achieve heart regeneration has emerged as a promising strategy against heart failure post‐ischemic injury [2]. Although recent advances have identified numerous pro‐regenerative targets [3, 4, 5, 6, 7, 8, 9, 10, 11, 12, 13, 14, 15], the field currently faces a critical bottleneck due to the lack of standardised methodologies for quantitatively assessing regenerative outcomes. This significantly impedes both therapeutic target identification and cross‐study comparisons.

Current assessment of heart regeneration in murine models relies on multiple parameters including echocardiographic evaluation of cardiac function, histological staining for fibrosis, and cardiomyocyte proliferation analysis. However, each of these approaches is subject to important limitations. Echocardiographic measurements are sensitive to operational variables such as anaesthesia depth, animal size, and transducer angle. Fibrosis evaluation via Masson's trichrome staining involves complex procedures with results highly dependent on technical execution. Proliferation assessment through immunofluorescence presents challenges of high subjectivity and low reproducibility across processing, staining, and interpretation stages. Meanwhile, cardiomyocyte counting by tissue digestion suffers from difficult‐to‐control enzymatic processes risking substantial cell loss, coupled with error‐prone subjective quantification. Collectively, these issues compromise experimental reproducibility and hinder robust preclinical validation.

In this study, we developed an integrated and standardised framework for the systematic evaluation of adult heart regeneration. By combining optimised echocardiographic acquisition under controlled physiological conditions with uniform histological, proliferative, and cardiomyocyte quantification strategies, this framework minimises inter‐operator variability and enables robust assessment of both functional recovery and structural regeneration. Importantly, the use of multi‐level fibrosis analysis, multi‐marker cell‐cycle readouts, and absolute cardiomyocyte counting provides a comprehensive and reproducible benchmark for evaluating regenerative outcomes across studies. Together, this platform establishes a reliable foundation for comparative heart regeneration research and therapeutic development.

## 2 Materials and Methods

### 2.1 Ethics Statement

Animal care and use for this study was approved by the Laboratory Animal Ethics Committee of Fuwai Hospital and conducted in accordance with the regulations outlined in Beijing Experimental Animals Management Regulations as well as the Guide of NIH for the Care and Use of Laboratory Animals. The ethical approval certificate number is 0108‐7‐4000‐ZX(X)‐042.

### 2.2 Animals

*C57BL/6* mice were purchased from Vital River Laboratory Animal Technology Co. Ltd. (Beijing, China). *Myh6‐MerCreMer* (JAX 005657) mice were acquired from the Jackson Laboratory (Bar Harbour, ME, USA). *MADM‐ML‐11* ^*GT/TG*^ mice were generously supplied by Dr. Jinghai Chen at the Institute of Translational Medicine, Zhejiang University (Hangzhou, China). All mice were maintained in individually ventilated cages, at 23°C ± 1°C and a relative humidity of 50% ± 5% with controlled illumination (12‐h dark/light cycle). Mice were given ad libitum access to food and water. Adult mouse models (aged 8–12 weeks) were used to study heart regeneration following ischemic cardiac injury.

### 2.3 Echocardiography

Cardiac function was determined by echocardiography at the designated time points using a digital ultrasound system (Vevo 3100 Imaging System, Visual Sonics, Toronto, Canada). For systolic function assessment, B‐mode ultrasound videos and M‐mode images were obtained from a parasternal short‐axis view at the papillary muscle level. Cardiac function was quantitatively evaluated using the software Vevo LAB (Version 5.7.1, Visual Sonics, Toronto, Canada). Systolic function parameters, including ejection fraction (EF) and fractional shortening (FS), were automatically calculated after manual tracing of the anterior and posterior walls using electronic callipers. 5–10 representative contraction cycles were analysed for each mouse. All echocardiographic measurements were performed in a blinded manner. To ensure statistical robustness and the reproducibility of findings, a minimum sample size of *n* = 6 biological replicates per group is recommended.

### 2.4 Tissue Harvesting and Embedding

Hearts were first collected and fixed at room temperature for 24–48 h in 4% paraformaldehyde (PFA, LEAGENE, DF0135), depending on tissue size. After fixation, the PFA was washed off with running tap water for 1 h. Dehydrated the hearts of adult mice through graded ethanol: 70% ethanol (30 min) → 80% ethanol (30 min) → 90% ethanol (2 changes, 30 min each) → 95% ethanol (2 changes, 30 min each) → 100% ethanol (2 changes, 8 min for the 1st time, and 10 min for the 2nd time). Submerged the cassettes for 8 min in xylene for clearing, and repeated once. Then, bathed the cassettes in melted paraffin for 30 min to replace xylene with wax, and repeated twice. Embeded the samples into paraffin blocks, apex upward and transverse section downward, using the embedding and cooling workstation (Thermo Scientific HistoStar, ThermoFisher, Waltham, MA, US).

### 2.5 Tissue Sectioning and Rehydration

For Masson's trichrome staining, paraffin sections were performed with a thickness of 5 μm, with the hearts sliced continuously from the ligation site to the apex every 200–300 μm. For assessment of cardiomyocyte proliferation, selected the region with the largest transverse cross‐sectional area within the ischemic zone for sectioning. Allowed sections to air‐dry completely at room temperature for 1 day, ensuring no visible moisture remains. Bake the tissue sections at 68°C for 45 min. Then immerse the slides in xylene (3 changes, 10 min each), then rehydrate with decreasing ethanol concentrations: 100% ethanol (2 changes, 5 min each) → 90% ethanol (5 min) → 80% ethanol (5 min) → 70% ethanol (5 min), followed by a 5‐min running tap water rinse.

### 2.6 Masson's Trichrome Staining

The slides were immersed in a 2.5% potassium dichromate (Sigma‐Aldrich, P5271) solution for an overnight period (12–18 h) at room temperature, which could enhance the affinity of the tissue to the dye and optimise the dyeing effect. Then the tissue sections were rinsed with running tap water. Masson's trichrome staining was then performed using a Masson's Trichrome Stain Kit (Sigma‐Aldrich, HT15), according to the manufacturer's protocol. Brightfield scanning of the entire heart tissue section was performed at 20× magnification to generate high‐resolution digital images using a slide digital scanner (Axio Scan Z1, Carl Zeiss Inc., Jena, Germany). Quantification of the fibrotic scar size was carried out using ImageJ software (Version 1.51, National Institutes of Health, USA). To ensure statistical robustness and the reproducibility of findings, a minimum sample size of *n* = 6 biological replicates per group is recommended.

### 2.7 5‐Ethynyl‐2′‐Deoxyuridine (EdU) Incorporation Assay

The solution of EdU powder (MCE, HY‐118411) was prepared as follows: For each 1 mL of solution, dissolve 1 mg of EdU powder in sterile saline (0.9% w/v) under thorough vortexing to ensure complete dissolution. The prepared EdU solution should be protected from light and stored at 4°C for up to 1 week. EdU was administered via intraperitoneal injection to mice at a dose of 10 mg/kg every 24 h until the time point of cardiomyocyte proliferation assessment. The assessment time point was selected based on the interval after intervention when significant changes occur, such as 3 or 7 days following pro‐regenerative factor stimulation. To ensure statistical robustness and the reproducibility of findings, a minimum sample size of *n* = 3 biological replicates per group is recommended.

### 2.8 Immunofluorescence

After rehydration, paraffin‐embedded tissue sections underwent antigen retrieval using EDTA‐based buffer (ZSGB‐Bio, ZLI‐9067; pH = 9.0) in a pressure cooker (121°C for 3 min). Then released the pressure from the cooker, left the sections inside the pot, and cooled them under running water for at least 20 min. For EdU staining, prepared the working solution (taking 1 mL total volume as an example) as follows: 879 μL Phosphate‐buffered saline (PBS, Solarbio, P1020) + 20 μL CuSO_4_ (100 mM, Sigma‐Aldrich, 209,198) + 100 μL L‐Ascorbic acid (0.5 M, Sigma‐Aldrich, A4403) + 1 μL EdU dye (2 mM, Thermo Fisher Scientific, A10277). Applied the staining solution to cover the tissue sections completely for 30 min at room temperature, protected from light, and then washed with PBS (Solarbio, P1020). Permeabilized the sections with 0.3% Triton X‐100 (Sigma‐Aldrich, T8787) and blocked with 5% normal donkey serum (Solarbio, SL050) in PBS (Solarbio, P1020) for 1 h. Primary antibodies were applied overnight at 4°C. After washing with PBS (Solarbio, P1020) three times, the slides were incubated with appropriate fluorescence‐labelled secondary antibodies for 1 h at room temperature. The following primary antibodies and reagents were used: Ki67 (Abcam, Ab16667; 1:500), phospho‐histone H3 (Millipore, 3,527,703; 1:500), Aurora B (Abcam, ab239837; 1:500), and cardiac troponin T (cTnT, DSHB, CT3, 1:200) antibodies. Secondary antibodies included: donkey anti‐rabbit Alexa Fluor 488 (Invitrogen, A‐21206; 1:400), donkey anti‐mouse Alexa Fluor 594 (Invitrogen, A‐21203; 1:400). After washing with PBS (Solarbio, P1020), the slides were mounted in Fluoroshield with DAPl (Sigma‐Aldrich, F6057) shielded from light. Scanned the entire heart tissue section using a fluorescence slide scanner (BZ‐X800, Keyence, Osaka, Japan) to obtain full‐section fluorescence images. Captured representative images using a confocal laser scanning microscope (LSM800, Carl Zeiss Inc., Jena, Germany). To ensure statistical robustness and the reproducibility of findings, a minimum sample size of *n* = 3 biological replicates per group is recommended.

### 2.9 The Mosaic Analysis for Double Markers (MADM) Study

For MADM clonal analysis, tamoxifen (Sigma‐Aldrich, T5648) was administered via intraperitoneal injection at a dose of 20 mg/kg body weight daily for 7 consecutive days, starting 2 weeks prior to injury. The mice hearts were harvested 14 days after ischemic injury surgery. Heart tissues were prepared as cryosections and stained with wheat germ agglutinin (WGA) conjugated to Alexa Fluor 647 (Invitrogen, W32466; 1:500) for 1 h at room temperature, and mounted in Fluoroshield with DAPl, shielded from light. Fluorescence images were acquired using a Zeiss LSM800 confocal laser scanning microscope (Carl Zeiss Inc., Jena, Germany). For quantitative analysis, the proportion of distinctly green‐ or red‐labelled cardiomyocytes relative to the total number of cardiomyocytes was calculated. To ensure statistical robustness and the reproducibility of findings, a minimum sample size of *n* = 3 biological replicates per group is recommended.

### 2.10 Enzymatic Digestion and Counting of Adult Cardiomyocytes

Adult hearts were harvested at the endpoint of echocardiographic functional assessment following ischemic injury. Immediately after collection, hearts were fixed in 4% PFA (LEAGENE, DF0135) at room temperature for 1 h. Following fixation, the 4% PFA solution was aspirated and discarded. Using sterile forceps, each heart was transferred to a fresh 5‐mL EP tube and washed three times with sterile PBS (Solarbio, P1020), each time incubating for 5 min with gentle agitation to remove residual fixative. Prepared 3 mL of the enzymatic digestion working solution per heart. The volume should be increased proportionally based on the number of hearts in the experiment. Taking 1 mL as an example, prepare 1 mL sterile PBS (Solarbio, P1020) + 1.8 mg Collagenase B (Roche, 11,088,831,001) + 2.4 mg Collagenase D (Roche, 11,088,858,001) + 100 μg Ampicillin (Beyotime Biotechnology, ST007). Cut each heart into 3‐mm pieces and transfer to a 3‐mL enzymatic digestion working solution. Digestion was performed at 37°C on an end‐over‐end shaker. Every 24 h, the enzyme solution containing dissociated cardiomyocytes was collected into a 15‐mL conical tube. Fresh enzyme solution was replenished to the remaining tissue in the 5‐mL EP tube. This collection‐replenishment cycle was repeated until no additional cardiomyocytes could be liberated from the tissue. Pooled enzyme solutions were filtered through a 160‐μm nylon mesh (Millipore, NY6H04700) to collect supernatants. The filtered cell suspension was gently homogenised by pipetting (wide‐bore tips) to ensure uniformity. A 15‐μL aliquot of the cell suspension was aspirated and transferred to a Countstar cell counting chamber (Countstar, CO010101). The Countstar cell counting chamber was scanned using a fluorescence microscope (BZ‐X800, Keyence, Osaka, Japan). The scanned images were analysed using the “Counting” plugin of ImageJ software (Version 1.51, National Institutes of Health, USA) for cardiomyocyte counting. To ensure statistical robustness and the reproducibility of findings, a minimum sample size of *n* = 3 biological replicates per group is recommended.

## 3 Results

### 3.1 Serial Echocardiographic Mapping Enables Precise Evaluation of Systolic Recovery

**FIGURE 1:**
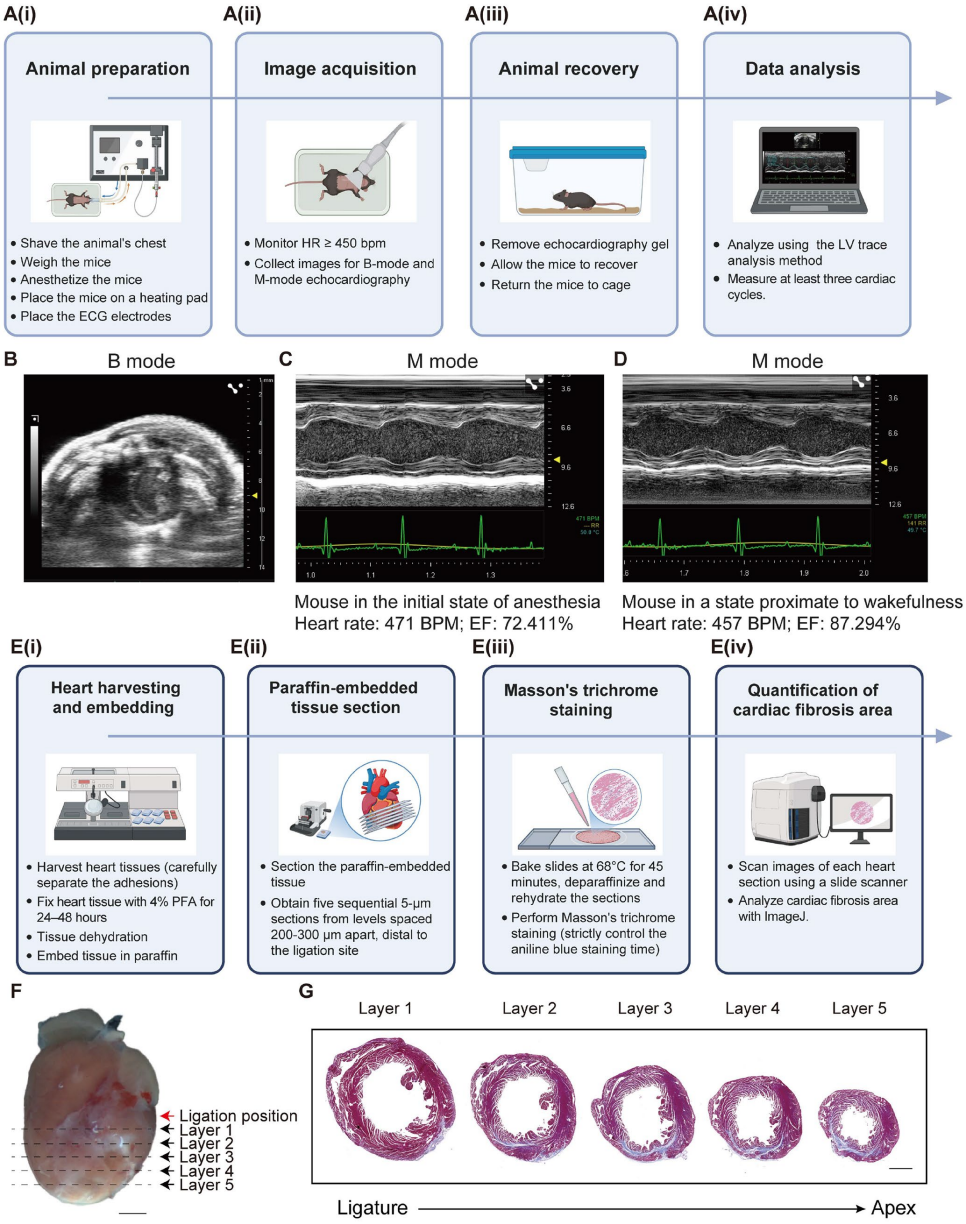
Evaluation of cardiac function and fibrosis. (A) The schematic diagram of assessment methods of cardiac systolic function by echocardiography, including the preparation for the detection (A(i)), the detection of cardiac systolic function (A(ii)), animal recovery (A(iii)) and data analysis (A(iv)). (B) A representative B‐mode echocardiographic image of the cardiac short‐axis view during systolic function assessment. (C, D) Representative M‐mode echocardiographic images and analysis of the same mouse under initial anaesthesia (C) and near‐awakening conditions (D). (E) The schematic diagram of assessment methods of cardiac fibrosis area by Masson's trichrome staining, including hearts harvesting and embedding (E(i)), paraffin‐embedded tissue section (E(ii)), Masson's trichrome staining (E(iii)) and quantification of cardiac fibrosis area (E(iv)). (F) A representative image illustrating the ligation site and section plane of cardiac tissue collected 56 days after ischemia/reperfusion (I/R) injury. (G) Representative Masson's trichrome staining images of paraffin‐embedded heart sections at different layers, collected 56 days after I/R injury.

To enable precise and reproducible assessment of mouse cardiac function, we established a standardised echocardiographic acquisition procedure that systematically integrates four key components, including animal preparation, image acquisition, post‐imaging recovery, and data analysis workflows (Figure 1A,B). This comprehensive framework minimises inter‐operator variability while ensuring ethical compliance and maximal data fidelity throughout the experimental pipeline.

First, to minimise confounding factors, ensure animal welfare, and enhance data reliability, we conducted meticulous animal preparation encompassing hair removal, anaesthesia stabilisation, positioning, and coupling application. Thoracic hair was removed using depilatory cream, a step best performed 1 day before imaging to minimise handling stress. For induction anaesthesia, we administered 5.0% isoflurane at 3.0–3.5 L/min via an induction chamber. Once anaesthetic depth was confirmed (loss of righting reflex and regular breathing), the mouse was then transferred to the imaging platform and positioned supine with limbs gently secured to ECG electrodes using adhesive tape. For maintaining anaesthesia, we used a nose cone with 1.0%–2.0% isoflurane at 0.5–1.0 L/min oxygen flow. The stabilisation of anaesthesia was crucial to the success of the experiment. In this step, we needed to ensure two critical points. One was to avoid imaging acquisition during the early phase of anaesthesia maintenance, due to the possible occurrence of transient myocardial depression, marked by a temporary reduction in ejection fraction despite a stable heart rate around 450 beats per minute (bpm). This transient dysfunction likely reflected anaesthetic‐induced myocardial stunning rather than a true cardiac impairment, and it typically resolved within 5 min (Figure 1C,D). To ensure measurement accuracy, we recommend stabilising the animal under reduced isoflurane (0.5 L/min) for 5 min before imaging began allowing for hemodynamic equilibrium, and data acquisition was initiated when early signs of consciousness reappeared. Another point was to maintain body temperature at 36°C–38°C, heart rate above 450 bpm, and respiration at 80–120 bpm for accurate echocardiographic imaging. We recommended targeting sustained heart rate (HR) > 500 bpm with comparable profiles across experimental groups, as HR < 450 bpm indicated cardiac depression [16]. A thin, bubble‐free layer of ultrasound coupling gel was then applied to the thorax.

After the animal preparation was completed, we proceeded with image acquisition of parasternal views. For the long‐axis (PLAX) view, the platform was tilted approximately 30° (head‐up position) while the transducer was positioned parallel to the long body axis, rotated 30° counterclockwise, and lowered diagonally from the left thorax while maintaining a 1–2 mm gel standoff to prevent cardiac compression which could lead to inaccurate measurements. In B‐mode, adjustments were made until the aortic root, ascending aorta, and left ventricular (LV) apex were clearly visible with the LV centered and the base‐to‐apex axis parallel to the transducer surface. After saving 5–10 cardiac cycles, M‐mode acquisition was performed with the sampling line perpendicular to the LV posterior wall at the papillary muscle level. Then, we moved on to acquire images of the short‐axis (PSAX) view by rotating the transducer 90° clockwise from the PLAX view and optimizing until a round LV contour was achieved with simultaneous visualization of both papillary muscles in the antero‐lateral and postero‐medial quadrants of the heart, respectively (Figure 1B). M‐mode acquisition in this view positioned the sampling line at the LV center.

Following imaging, we gently removed the coupling gel with soft tissue paper from the mouse chest, allowed the animals to recover from the anaesthesia on a heating pad, and then returned them to the warm breeding cage when they awakened.

For data analysis, we utilised the M‐mode tracing method by Vevo LAB's LV Trace tool to track endocardial and epicardial borders over 3–5 consecutive cardiac cycles for automated parameter calculation. Alternatively, it was suggested to mark the left ventricular interventricular septum thickness at end‐diastole and end‐systole (IVS; d, IVS; s) each for 3 times, and calculate the average values to obtain EF and FS values. We summarised key cardiac functional indicators commonly used in heart regeneration research. EF was the most frequently adopted parameter for evaluating left ventricular systolic function [3, 4, 5, 6, 7, 8, 9, 10, 11, 12, 13, 17, 18, 19]. However, given the pathological remodelling and irregular ventricular geometry following ischemic injury, ventricular function was assessed using multiple complementary metrics, including FS, left‐ventricular end‐diastolic anterior wall thickness (LVAW; d), left‐ventricular end‐systolic anterior wall thickness (LVAW; s), left‐ventricular end‐diastolic posterior wall thickness (LVPW; d), left‐ventricular end‐systolic posterior wall thickness (LVPW; s), left‐ventricular end‐diastolic internal diameter (LVID; d), and left‐ventricular end‐systolic internal diameter (LVID; s) (Table 1) [20]. Troubleshooting advice can be found in Table 2.

**TABLE 1:** Key parameters from left ventricular long‐ and short‐axis.

	Abbreviation	Full name	Definition
Left ventricular systolic function parameters	EF	Ejection fraction	Percentage of blood ejected from the left ventricle per beat
	FS	Fractional shortening	Percentage reduction in left ventricular diameter during systole
	SV	Stroke volume	Volume of blood ejected per beat. Calculation: End‐diastolic volume – End‐systolic volume
	CO	Cardiac output	Blood volume pumped by the heart per minute. Formula: SV × HR
	LVEDV	Left ventricular end‐diastolic volume	Volume of blood in the left ventricle at end‐diastole
	LVESV	Left ventricular end‐systolic volume	Volume remaining at end‐systole
Left ventricular structure parameters	LVAW; d	Left ventricular anterior wall thickness at end‐diastole	Thickness of the left ventricular anterior wall during diastole
	LVAW; s	Left ventricular anterior wall thickness at end‐systole	Thickness during systole; typically slightly thinner than diastolic values
	LVPW; d	Left ventricular posterior wall thickness at end‐diastole	Thickness of the posterior wall during diastole
	LVPW; s	Left ventricular posterior wall thickness at end‐systole	Thickness during systole; used to assess regional wall motion abnormalities
	LVID; d	Left ventricular internal dimension at end‐diastole	Diameter of the left ventricle during diastole
	LVID; s	Left ventricular internal dimension at end‐systole	Diameter during systole

**TABLE 2:** Troubleshooting table.

Problem	Possible reason	Solution
Serial echocardiographic mapping enables precise evaluation of systolic recovery		
Low heart rate	Extensive anaesthesia	Reduce the isoflurane oxygen flow rate to ~0.5 L/min after inducing anaesthesia
Blurred image	Poor probe contact	Reapply the coupling agent
	Insufficient coupling agent	Adjust the probe pressure
Incomplete visualisation of the left ventricular cavity	Deviation in the sectional angle	Slowly rotate the probe and observe the dynamic images to adjust to the optimal section
Standardised fibrosis staining achieves quantitative and reproducible assessment of post‐ischemic scar burden		
Prone to tissue fracture when sectioning	Insufficient or excessive fixation time	Control fixation duration (24–48 h for routine tissues)
	Inadequate dehydration	Optimise ethanol gradient and xylene exposure
	Excessive wax temperature	Maintain wax at 58°C–62°C
Unclear nuclear staining	Aged Modified Lillie‐Mayer's Haematoxylin Solution	Replace with fresh Modified Lillie‐Mayer's Haematoxylin Solution
	Insufficient haematoxylin incubation time	Extend haematoxylin incubation time
	Excessive rinsing	Shorten the running water rinsing time
Dark cytoplasmic background	Excessive haematoxylin incubation time	Shorten the haematoxylin incubation time, or perform additional hydrochloric acid‐alcohol differentiation until complete cytoplasmic decolorization is attained
Poor differentiation between muscle fibres and collagen	Excessive staining time with Biebrich Scarlet‐Acid Fuchsin Solution	Shorten the staining time of Biebrich Scarlet‐Acid Fuchsin Solution
Too light staining of collagen	Insufficient aniline blue concentration	Increase the staining time of Aniline Blue Solution
Integrated multi‐marker profiling provides high‐resolution quantification of cardiomyocyte cell‐cycle reactivation		
Prone to tissue fracture when sectioning	Insufficient or excessive fixation time	Control fixation duration (24–48 h for routine tissues)
	Inadequate dehydration	Optimise ethanol gradient and xylene exposure
	Excessive wax temperature	Maintain wax at 58°C–62°C
Poor tissue morphology after sectioning	Blunt microtome blade or incorrect cutting angle	Replace blade regularly and ensure proper blade inclination
Tissue detachment from slides	Slides not properly coated	Use positively charged or adhesive slides
	Over‐heating during antigen retrieval	Ensure the pressure cooker has cooled adequately before opening
	Vigorous handling	Handle slides gently during washing
Weak or absent EdU signal	EdU solution degraded due to improper storage or prolonged storage	Prepare fresh EdU solution for injection, protect from light, and do not use beyond 1 week at 4°C
	“Click” reaction staining solution prepared incorrectly or degraded	Prepare the CuSO_4_‐Ascorbic Acid‐dye mixture immediately before use and keep it on ice, protected from light
No specific signal	Primary antibody failure	Validate antibodies on positive control tissue
	Antigen retrieval not optimal for the target epitope	Test different antigen retrieval buffers (e.g., citrate vs. EDTA) and methods
	Secondary antibody does not match primary antibody host species	Confirm the host species of primary antibodies and use appropriate secondary antibodies
	Insufficient incubation time or mismatch in excitation wavelength	Extend the incubation time (overnight at 4°C for primary antibody / 1 h at 37°C for secondary antibody), and check the compatibility of excitation wavelengths using a fluorescence microscope
High background noise	Inadequate blocking	Ensure blocking is performed with 5% normal serum (matching secondary antibody host) for full 1 h
	Primary antibody concentration too high	Titrate the primary antibody to find the optimal dilution
	Inadequate washing	Increase washing times and duration after antibody incubations
Absolute cardiomyocyte enumeration offers definitive metrics of regenerative efficacy in the adult heart		
Low cell pellet yield after filtration	Inefficient digestion due to enzyme (collagenase B/D) inactivation	Ensure enzymes are aliquoted and stored properly, and avoid repeated freeze–thaw cycles
	Over‐fixation by PFA	Limit PFA fixation time to 1 h to prevent over‐crosslinking
Excessive debris in cell suspension	Incomplete filtration	Ensure effective filtration through a sterile 160‐μm nylon mesh. After centrifugation, carefully resuspend the pellet and wash with PBS 1–2 times if needed
	Cell disintegration	Reduce the concentration of enzymes, or shorten the digestion time
Cells form clumps, uneven distribution	Rough pipetting causing shear stress or cell aggregation	Resuspend the cell pellet gently using wide‐bore pipette tips. Let the suspension stand briefly to allow large clumps to settle before sampling

### 3.2 Standardised Fibrosis Staining Achieves Quantitative and Reproducible Assessment of Post‐Ischemic Scar Burden

For systematic quantification of myocardial fibrosis in murine models, we developed a standardised histopathological workflow consisting of heart harvesting and embedding, paraffin‐embedded tissue sectioning, Masson's trichrome staining, and quantitative analysis of cardiac fibrosis area (Figure 1E).

To comprehensively evaluate myocardial fibrosis area, we standardized the tissue sampling and sectioning procedures for Masson's trichrome staining. After final functional assessment, we collected the hearts with particular attention to separating adherent cardiac tissue from the chest wall in ischemic models. Following fixation in 4% PFA for 24–48 h, tissues underwent graded ethanol dehydration, xylene clearing, and paraffin embedding. Systematic sectioning yielded five 5‐μm transverse levels at 200–300 μm intervals from the ligation site to the apex (Figure 1F), ensuring comprehensive representation of fibrotic changes.

Given the relatively complex procedures and the significant influence of operational factors on the stability of staining results, we established a standardised and detailed operating procedure for Masson staining. Following the tissue sectioning and rehydration, we immersed the slides in a 2.5% potassium dichromate solution overnight at room temperature to enhance the affinity of the tissue to the dye and rinsed the tissue sections with running tap water. For nuclei staining, the slides were incubated in Modified Lillie‐Mayer's Haematoxylin Solution for 5 min and differentiated in 1% hydrochloric acid‐alcohol for 3–5 s. Optimal staining was achieved when nuclei appeared blue and cytoplasm remained colourless. For cytoplasm staining, the slides are incubated in Biebrich Scarlet‐Acid Fuchsin Solution for 5 min and differentiated in Phosphotungstic/Phosphomolybdic Acid Solution for 5 min, with optimal staining characterised by red‐stained cytoplasm and pale pink collagen fibres. After staining of nuclei and cytoplasm, we continued with collagen fibre staining by incubating the slides in Aniline Blue Solution for 10–30 s. This step required strict control of the staining time and thus it was recommended to stain only one slide at a time. Collagen fibres were supposed to be distinct blue while the cytoplasm remained red. In this staining procedure, it was essential to confirm optimal staining and consistency under microscopic examination after each step as the quality control measures. Then, we dehydrated the tissue sections through 100% alcohol, clear in xylene, and sealed with a coverslip using a mounting medium.

Following staining, we performed brightfield scanning of the entire heart tissue section at 20× magnification to generate high‐resolution digital images (Figure 1G). Fibrotic area was quantified using ImageJ with Colour Deconvolution, calculating the percentage of collagen fibre area relative to the total left ventricular area across all sections. Troubleshooting guidance is provided in Table 2.

### 3.3 Integrated Multi‐Marker Profiling Provides High‐Resolution Quantification of Cardiomyocyte Cell‐Cycle Reactivation

**FIGURE 2:**
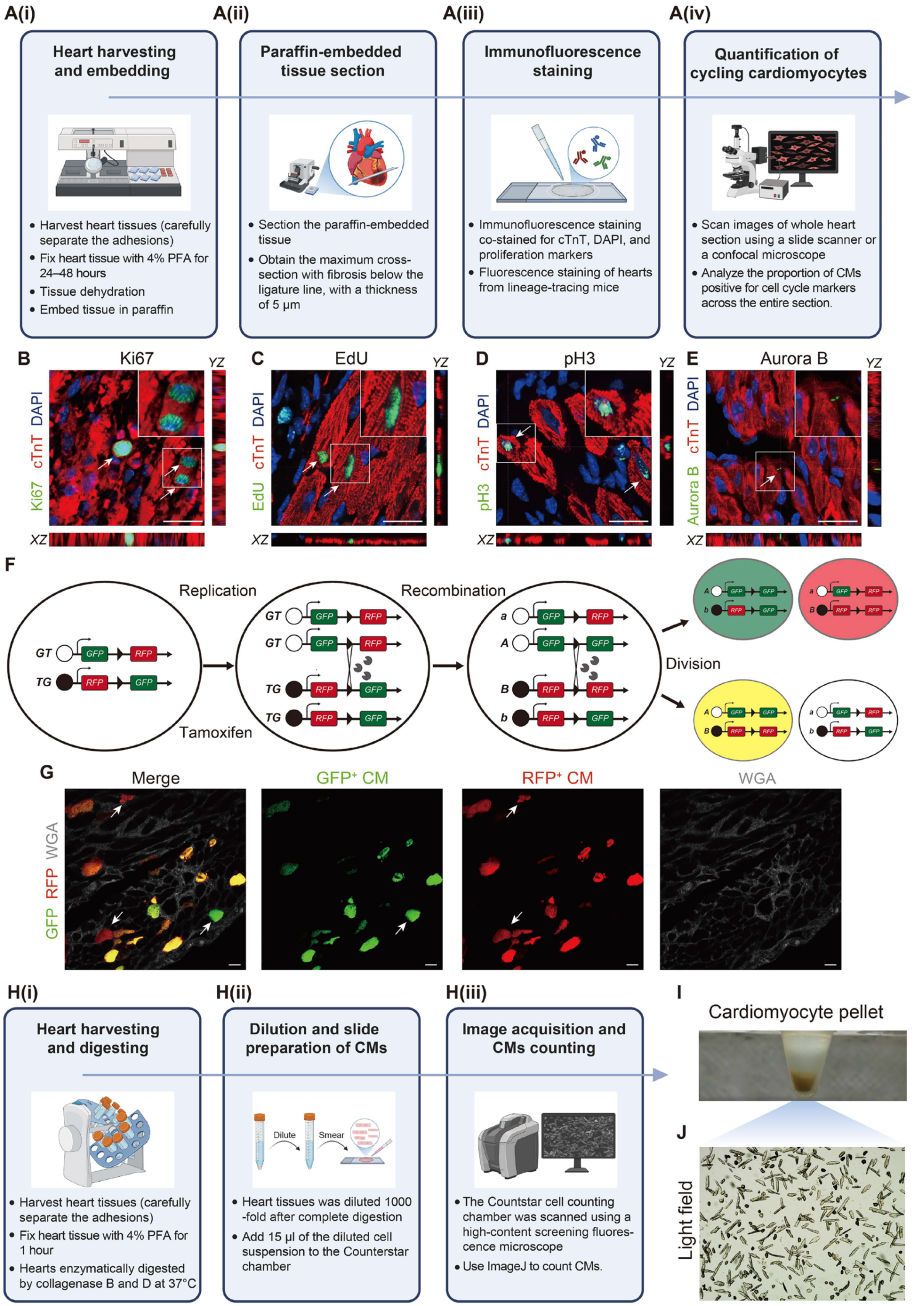
Cardiomyocyte proliferation detection and quantitative counting. (A) The schematic diagram of cycling cardiomyocytes by immunofluorescence staining, including hearts harvesting and embedding (A(i)), paraffin‐embedded tissue section (A(ii)), immunofluorescence staining (A(iii)) and quantification of cycling cardiomyocytes (A(iv)). (B) The schematic diagram of the cell cycle and proliferation markers. (C–E) Representative immunofluorescence staining images of Ki67^+^ (B), EdU^+^ (C), pH3^+^ (D) and Aurora B^+^ (E) cardiomyocytes. (F) Schematic of the MADM lineage tracing principle for assessing cardiomyocyte proliferation. (G) Representative fluorescence images of single‐labelled (red or green; arrowheads), double‐labelled (yellow), and unlabeled (without fluorescence) cardiomyocytes in MADM mice. (H) The schematic diagram of enzymatic dissociation of cardiomyocytes and total cardiomyocyte counting, including hearts harvesting and digesting (H(i)), dilution and slide preparation of cardiomyocytes (H(ii)) and images acquisition and cardiomyocytes counting (H(iii)). (I) Complete dissociation of cardiomyocytes yields a pellet of digested cardiomyocyte. (J) Representative light field images of isolate cardiomyocytes. CM indicates cardiomyocyte.

To resolve the methodological ambiguity in immunofluorescent staining of cell cycle markers, we developed a standardised multi‐parametric immunofluorescence strategy that evaluates four essential cell‐cycle markers: Ki67 for overall cell‐cycle entry, EdU incorporation for S‐phase detection, phospho‐Histone H3 (Ser10) (pH3) for mitotic entry, and Aurora B kinase localization for cytokinesis completion in ischemic‐injured adult mouse hearts (Figure 2B–E). This integrated methodology offered exceptional resolution for tracking cardiomyocyte cell‐cycle progression from initial activation through DNA synthesis and cytokinesis.

First, we administered EdU daily via intraperitoneal injection (10 mg/kg body weight) until the endpoint assessment at 7 days post‐injury, ensuring consistent labeling of DNA‐synthesising cells. Following collection, hearts were fixed in 4% PFA for 24–48 h, processed through graded ethanol dehydration and xylene clearing, and embedded in paraffin. Then, we obtained 5‐μm tissue sections of the maximal cross‐sectional area from the ischemic zone beneath the ligation site.

Given that adult cardiomyocytes were terminally differentiated cells exhibiting limited regenerative potential, specialised morphology (e.g., elongated rod shape, intercalated discs), improper sample processing may disrupt cytoskeletal structures or intercellular junctions, compromising staining quality. Therefore, we provided a detailed cardiomyocyte‐specific sample staining procedure as follows. For antigen retrieval, paraffin‐embedded tissue sections were immersed in EDTA antigen retrieval buffer in a pressure cooker (pH = 9.0), and maintain boiling for 2 min. Then the pressure was released from the cooker, the sections were left inside the pot, and cooled under running water for at least 20 min. For EdU staining, the slides were incubated in EdU staining working solution for 30 min at room temperature, protected from light. The sections were permeabilized with 0.3% Triton X‐100 and blocked with 5% normal donkey serum in PBS for 1 h. Then we incubate primary antibody overnight at 4°C in validated concentration (cTnT 1:200, Ki67 1:500, pH3 1:500, Aurora B 1:500). After washing with PBS three times, the slides were incubated with appropriate fluorescence‐labelled secondary antibodies for 1 h at room temperature, and mounted in Fluoroshield with DAPl, shielded from light.

For image acquisition, we recommended scanning the entire heart tissue section using a fluorescence slide scanner to obtain full‐section fluorescence images, and capturing representative images using a confocal laser scanning microscope in Z‐stacks mode. In this step, it was critical to distinguish cardiomyocytes and non‐cardiomyocytes. Cardiomyocytes must exhibit a complete perinuclear cTnT staining forming a distinct sarcomeric lattice. Cardiomyocytes were classified as EdU‐positive, Ki67‐positive or pH3‐positive only when the respective signals exhibit complete colocalization with cardiomyocyte nuclei (Figure 2B–D). Aurora B‐positive cardiomyocytes had to display punctate or midbody‐associated linear morphologies confined to the intercellular cleavage plane between two adjacent cardiomyocytes (Figure 2E). Troubleshooting advice can be found in Table 2.

### 3.4 Using the MADM System for Assessing Cardiomyocyte Proliferation In Vivo

To provide permanent genetic proof of completed cytokinesis, the MADM system offers an unparalleled lineage‐tracing framework that moves beyond snapshots. By utilising Cre‐mediated interchromosomal recombination during the G2 phase of the cell cycle, the MADM system concurrently labels daughter cells with distinct fluorescent reporters (e.g., GFP and RFP), where the appearance of “twin spots” (Figure 2F), physically adjacent, monochromatic green and red cells, serves as unambiguous genetic evidence of a completed division event. *MADM‐ML‐11* ^*GT/TG*^ mice were crossed with the *Myh6‐MerCreMer* mice to generate experimental progeny. To induce Cre‐mediated recombination, tamoxifen (Sigma‐Aldrich, T5648) was administered via intraperitoneal injection at a dosage of 20 mg/kg body weight once daily for 7 consecutive days. This induction protocol was initiated 14 days prior to the injury to ensure stable clonal labeling and sufficient tamoxifen clearance before the experimental stimulus. After a defined chase period (generally 14 days or longer post‐injury), hearts were harvested and processed into cryosections to preserve the spatial architecture of the labelled clones. Sections were then stained with WGA conjugated to Alexa Fluor 647 for 1 h at room temperature, mounted in Fluoroshield containing DAPI, and protected from light. Subsequent confocal imaging allows for the precise quantification of these events, where the proliferative capacity is statistically evaluated by calculating the percentage of monochromatic (either red‐only or green‐only) cardiomyocytes relative to the total number of cardiomyocytes (Figure 2G), establishing a rigorous, single‐cell resolution metric for evaluating true regenerative capacity in the adult heart.

### 3.5 Absolute Cardiomyocyte Enumeration Offers Definitive Metrics of Regenerative Efficacy in the Adult Heart

For precise control of cardiac tissue digestion conditions and accurate cardiomyocyte counting, we developed a standardised enzymatic digestion method for the isolation and enumeration of cardiomyocytes from adult mouse hearts, enabling conclusive assessment of regenerative outcomes (Figure 2H).

To begin with, we collected the heart tissues after final functional evaluation. Harvested tissues were immediately fixed in 4% PFA for 1 h at room temperature to maintain cellular integrity, followed by PBS washing. Our enzymatic digestion condition involved mincing each heart into 3 mm^3^ fragments, which were then incubated in 3 mL of digestion buffer containing Collagenase B (1.8 mg/mL), Collagenase D (2.4 mg/mL), and ampicillin (100 μg/mL). Subsequently, we digested the tissue fragments at 37°C under continuous rotation, with solution collection and enzyme replenishment every 24 h for 2–3 cycles. Alternative approaches including Langendorff and non‐Langendorff perfusion techniques had also been employed for cardiomyocyte isolation and quantification [21, 22], though the present enzymatic method offered particular advantages for large‐scale processing.

Following digestion, we filtered the cell suspension through 160‐μm nylon mesh and concentrated by low‐speed centrifugation (500 rpm, 2 min). After gentle homogenization and 1000‐fold dilution, 15‐μL aliquots were loaded into Countstar chambers for bright‐field scanning at 10× magnification (Figure 2I,J).

For image analysis and quantitative counting, we used the “Cell Counter plugin” of ImageJ. Only intact rod‐shaped cardiomyocytes were included in the statistical analysis to minimise errors. The absolute counts of cardiomyocytes were calculated as: Cardiomyocyte number = Counted cells × Dilution factor. Troubleshooting advice can be found in Table 2.

## 4 Discussion

Heart regeneration has been described as the holy grail of cardiology and is defined as the replacement of lost or damaged myocardium with newly generated, fully functional cardiomyocytes [2]. True heart regeneration after injury can be rigorously defined by the following criteria: the completion of cardiomyocyte cytokinesis, which leads to a net increase in the total number of contractile cardiomyocytes and thereby drives the restoration of cardiac function together with a concomitant reduction in fibrotic tissue formation [2, 23, 24]. The evaluation of heart regeneration following myocardial ischemic injury in an adult mouse model has long been constrained by the lack of systematic and standardised methodologies for outcome assessment, which substantially limits both therapeutic target validation and cross‐study comparability. To address this challenge, we established an integrated evaluation framework by standardising and optimising four key analytical dimensions: echocardiographic functional assessment, fibrosis quantification, multi‐marker proliferation analysis, and absolute cardiomyocyte counting. This methodological system provides a reproducible, quantitative, and cross‐compatible platform for evaluating regenerative outcomes. Its establishment not only addresses the current methodological bottleneck in the field but also offers a critical technical foundation for the future preclinical translation of cardiac regenerative therapies.

Echocardiography is widely employed as the preferred method for evaluating cardiac function in rodents, owing to its non‐invasiveness, safety, reproducibility, broad accessibility, and low cost [25]. During the experiments, we observed that some mice exhibited pronounced transient myocardial depression during early maintenance anaesthesia. Therefore, we recommend maintaining a low‐dose anaesthesia for ~5 min, and then conducting the examination when the mice are about to regain consciousness. Furthermore, we summarise key cardiac functional indicators commonly used in heart regeneration research. EF is the most frequently adopted parameter for evaluating left ventricular systolic function [3, 4, 5, 6, 7, 8, 9, 10, 11, 12, 13, 17, 18, 19]. In various studies on heart regeneration, mice with modelled cardiac ischemic injury showed a recovery range of 13.5%–42.3% in EF after regenerative therapy compared to control groups (Table 3). Moreover, to elucidate the efficacy of targeted interventions in promoting heart regeneration post‐ischemic injury, we proposed longitudinal monitoring of left ventricular systolic performance. The timeline for serial assessments is outlined in Table 4, with key evaluation nodes strategically selected to capture both early‐phase adaptations and long‐term functional remodelling. Notably, validating therapeutic factor‐induced heart regeneration requires meeting two efficacy endpoints: (i) Terminal Efficacy: EF at the terminal time point must show statistically significant improvement in the treatment group versus controls (*p* < 0.05); (ii) Dynamic Recovery: Serial echocardiography must document that terminal EF values in the treatment group significantly exceed their post‐injury nadir (△EF ≥ 10% with *p* < 0.05). These criteria, when corroborated by cardiomyocyte proliferation markers, provide robust evidence for regenerative efficacy. Conversely, if only terminal EF superiority over controls is observed without significant temporal recovery from the post‐injury nadir, the factor should be characterised as anti‐remodelling rather than pro‐regenerative. All analyses must be conducted blinded to group assignments to ensure objective assessment.

**TABLE 3:** Evaluation of heart regeneration studies.

Model	Pro‐regenerative factor	△Scar area (Control − Treatment)	△EF (Treatment − Control)	Fold change and ratio of proliferative positive cardiomyocytes (Treatment vs. Control)	Reference
I/R	Thyroid hormone	**9.5%** (*Thra* ^*DN/+*^ vs. *Myh6‐Cre; Thra* ^*DN/+*^, *P* < 0.01)	**42.3%** (*Myh6‐Cre; Thra* ^*DN/+*^ vs. *Thra* ^*DN/+*^, *P* < 0.0001)	Ki67^+^ cardiomyocyte (remote): **4.9** (0.49% *Myh6‐Cre; Thra* ^*DN/+*^ vs. 0.1% *Thra* ^*DN/+*^, *P* < 0.05)	*Science*. 2019;364 (6436):184–188.
				Ki67^+^ cardiomyocyte (border): **11.5** (2.3% *Myh6‐Cre; Thra* ^*DN/+*^ vs. 0.2% *Thra* ^*DN/+*^, *P* < 0.01)	
				EdU^+^ cardiomyocyte: **4.0** (0.16% *Myh6‐Cre; Thra* ^*DN/+*^ vs. 0.04% *Thra* ^*DN/+*^, *P* < 0.05)	
				pH3^+^ cardiomyocyte (remote): **8.5** (0.02% *Myh6‐Cre; Thra* ^*DN/+*^ vs. 0.17% *Thra* ^*DN/+*^, *P* < 0.05)	
				pH3^+^ cardiomyocyte (border): **12.8** (0.05% *Myh6‐Cre; Thra* ^*DN/+*^ vs. 0.64% *Thra* ^*DN/+*^, *P* < 0.001)	
I/R	Paromomycin (Paro) and neomycin (Neo)	**3.3%** (DMSO vs. Paro, *P* < 0.01); **4.8%** (DMSO vs. Paro‐Neo, *P* < 0.001)	**18.9%** (Paro vs. DMSO, *P* < 0.0001); **24.3%** (Paro‐Neo vs. DMSO, *P* < 0.0001)	pH3^+^ cardiomyocyte (remote): (0.01% Paro vs. 0.00% DMSO, *P* > 0.05); (0.03% Paro‐Neo vs. 0.00% DMSO, *P* < 0.001)	*Nat Cardiovasc Res*. 2024;3 (3):372–388.
				Aurora B^+^ cardiomyocyte (remote): (0.01% Paro vs. 0.00% DMSO, *P* > 0.05) (0.02% Paro‐Neo vs. 0.00% DMSO, *P* < 0.01)	
				pH3^+^ cardiomyocyte (border): (0.02% Paro vs. 0.00% DMSO, *P* > 0.05); (0.10% Paro‐Neo vs. 0.00% DMSO, *P* < 0.001)	
				Aurora B^+^ cardiomyocyte (border): (0.02% Paro vs. 0.00% DMSO, *P* > 0.05) (0.03% Paro‐Neo vs. 0.00% DMSO, *P* < 0.01)	
MI	*Ptma*	**14.7%** (AAV9‐*Luc* vs. AAV9‐*Ptma*, *P* < 0.01)	**25.4%** (AAV9‐*Ptma* vs. AAV9‐ *Luc*, *P* < 0.001)	EdU^+^ cardiomyocyte: **3.1** (0.22% AAV9‐*Ptma* vs. 0.07% AAV9‐*Luc*, *P* < 0.01)	*Sci Adv*. 2025;11 (21):eadt9446.
				pH3^+^ cardiomyocyte: **2.9** (0.16% AAV9‐*Ptma* vs. 0.06% AAV9‐*Luc*, *P* < 0.001)	
MI	*SphK2*	**27.6%** (AAV9‐GFP vs. AAV9‐*SphK2* + SphK1i, *P* < 0.01)	**27.9%** (AAV9‐*SphK2* + SphK1i vs. AAV9‐GFP, *P* < 0.05)	pH3^+^ cardiomyocyte: **4.0** (0.20% AAV9‐*SphK2* + SphK1i vs. 0.05% Saline, *P* < 0.05)	*Cell Metab*. 2024;36 (4):839–856.e8.
				Aurora B^+^ cardiomyocyte: **5.7** (0.17% AAV9‐*SphK2* + SphK1i vs. 0.03% Saline, *P* < 0.0001)	
MI	Versican	**15.2%** (PBS vs. Versican, *P* < 0.0001)	**18.5%** (Versican vs. PBS, *P* < 0.0001)	Ki67^+^ cardiomyocyte: **4.0** (0.08% Versican vs. 0.02% PBS, *P* < 0.01)	*Circulation*. 2024;149 (13):1004–1015.
				pH3^+^ cardiomyocyte: **2.5** (0.05% Versican vs. 0.02% PBS, *P* < 0.01)	
				Aurora B^+^ cardiomyocyte: **4.0** (0.008% Versican vs. 0.002% PBS, *P* < 0.001)	
MI	LIN28a	**9.5%** (WT vs. LIN28a, *P* < 0.01)	**25.0%** (LIN28a vs. WT, *P* < 0.001)	EdU^+^ cardiomyocyte: **2.2** (9.51% LIN28a vs. 4.25% WT, *P* < 0.05)	*Circulation*. 2023;147 (4):324–337.
				pH3^+^ cardiomyocyte: **1.4** (0.27% LIN28a vs. 0.19% WT, *P* < 0.001)	
MI	Five small molecules (5SM, Phenylephrine hydrochloride, Baricitinib, Harmine, Vo‐ohpic trihydrate and AZD3965)	**27.5%** (Control vs. 5SM, *P* < 0.01)	**26.8%** (5SM vs. Control, *P* < 0.0001)	Ki67^+^ cardiomyocyte: **3.5** (2.56% 5SM vs. 0.73% Control, *P* < 0.0001)	*Cell Stem Cell*. 2022;29 (4):545–558.e13.
				pH3^+^ cardiomyocyte: **2.2** (0.26% 5SM vs. 0.12% Control, *P* < 0.01)	
MI	gp130	**20.0%** (AAV9‐NC vs. AAV9‐*gp130* ^*ACT*^, *P* < 0.001)	**17.7%** (AAV9‐*gp130* ^*ACT*^ vs. AAV9‐NC, *P* < 0.05)	pH3^+^ cardiomyocyte (remote): **5.5** (0.11% AAV9‐*gp130* ^*ACT*^ vs. 0.02% AAV9‐NC, *P* < 0.05)	*Circulation*. 2020;142 (10):967–982.
				pH3^+^ cardiomyocyte (border): **11.0** (0.11% AAV9‐*gp130* ^*ACT*^ vs. 0.01% AAV9‐NC, *P* < 0.01)	
MI	Pkm2	**28.3%** (Luc K vs. _CMS_Pkm2, *P* < 0.01)	**15.2%** (_CMS_Pkm2 vs. Luc K, *P* < 0.0001)	pH3^+^ cardiomyocyte: **8.3** (0.33% _CMS_Pkm2 vs. 0.04% Luc K, *P* < 0.0001)	*Circulation*. 2020;141 (15):1249–1265.
MI	Hypoxia	**16.1%** (Normoxia vs. Hypoxia, *P* < 0.01)	**20.9%** (Hypoxia vs. Normoxia, *P* < 0.05)	pH 3^+^ cardiomyocyte: **4.3** (0.13% Hypoxia vs. 0.03% Normoxia, *P* < 0.01)	*Nature*. 2017;541 (7636):222–227.
MI	Agrin	**10.1%** (PBS vs. Agrin, *P* < 0.01)	**17.4%** (Agrin vs. PBS, *P* < 0.01)	Ki67^+^ cardiomyocyte: **12.5** (3.50% Agrin vs. 0.28% PBS, *P* < 0.05)	*Nature*. 2017;547 (7662):179–184.
				Aurora B^+^ cardiomyocyte: **7.6** (1.75% Agrin vs. 0.23% PBS, *P* < 0.05)	

*Note:* △Scar size (%) was the difference of fibrotic area between the pro‐regenerative factor treatment group and the control group at the final time point. △EF (%) value was the difference of EF between the final time point and post‐MI/I/R time point (such as 1 day post‐MI/I/R in the pro‐regenerative factor treatment group). Fold change of proliferation‐positive cardiomyocytes meant the ratio of cardiomyocytes positive for the proliferation markers overlapping with those used in our study (EdU, pH3, Aurora B) in the pro‐regenerative factor treatment group to that in the control group at the indicated time points. The statistical significance of the bold values has been added to the table.

Abbreviations: EdU, 5‐ethynyl‐2′‐deoxyuridine; EF, ejection fraction; I/R, ischemia/reperfusion; MI, myocardial infarction; pH3, phospho‐histone H3.

**TABLE 4:** Cardiac function assessment timeline and endpoint selection.

Time points	Assessment purpose
Pre‐operation (Baseline)	To establish individual reference values. Must be performed ≥ 24 h before surgery to avoid acute stress effects
Post‐operation Day 1	To validate surgical success and exclude perioperative mortality (mandatory). Confirm injury model effectiveness and document initial functional decline. For example, ejection fraction drops ≥ 30%–40% vs. baseline
Post‐operation Days 3, 7	To monitor early regenerative response (cardiomyocyte proliferation phase)
Post‐operation Days 14, 28	To evaluate long‐term functional recovery (remodelling phase)

*Note:* The selection of the endpoint depends on the therapeutic effect of different regenerative stimulating factors. Ideally, the cardiac function of the experimental group should be significantly higher than that of the control group, and also significantly higher than the lowest point of cardiac function after injury (the time point when the regenerative factor has not yet taken effect after the injury, such as 1 day or 7 days after the injury). In the best case, there will be no significant difference in cardiac function of the experimental group compared to the baseline.

Following ischemic injury, damaged myocardial tissue is often replaced by fibrous scars [26]. A key pathological hallmark of heart regeneration is the replenishment of injured cardiomyocytes, leading to a reduction in fibrotic scar area [8, 17, 18]. Masson's trichrome staining is the most common method for evaluating cardiac fibrosis [27]. Therefore, the accurate quantification of this metric through standardised Masson's trichrome staining is crucial for objectively evaluating the efficacy of heart regeneration therapies and elucidating the underlying mechanisms. Here, we present a standardised method for Masson's trichrome staining that encompasses the entire process from reproducible tissue sampling and sectioning to controlled staining and unbiased quantitative analysis, enabling reliable assessment of cardiac fibrosis after ischemic injury. Across various studies on heart regeneration, the observed reduction in fibrotic scar area shows a wide range, from 3.3% to 28.3%, compared to control groups (Table 3). This considerable variation can be attributed to factors such as differences in the ligation site of the animal models and the varying potency of the therapeutic interventions. These findings underscore the importance of standardised staining and quantification for accurately evaluating regenerative therapies. The methodology presented herein provides a reliable framework for assessing cardiac fibrosis, supporting consistent implementation across studies and facilitating valid comparisons of therapeutic efficacy in myocardial repair research.

Cardiomyocyte proliferation is the fundamental process underlying heart regeneration. The critical importance of accurately quantifying cardiomyocyte proliferation is underscored by clinical observations demonstrating that functional cardiac recovery correlates strongly with cardiomyocyte renewal capacity. In a landmark study, Bergmann and colleagues, using retrospective ^14^C birth‐dating analysis in patients with advanced heart failure, demonstrated a striking correlation between cardiomyocyte renewal capacity and cardiac functional recovery during left ventricular assist device (LVAD) support. Specifically, patients who exhibited improvement in left ventricular function (“responders”) showed a > 100‐fold increase in annual cardiomyocyte renewal rates compared to non‐responders and non‐LVAD patients [28]. This suggests that functional cardiac recovery may be intrinsically linked to a reactivation of endogenous cardiomyocyte regenerative potential in the adult human heart. Therefore, precise labeling and quantification of proliferating cardiomyocytes represent a core component of heart regeneration research.

Immunofluorescence staining of cell cycle markers serves as the most basic yet essential method for detecting cardiomyocyte proliferation [3, 4, 5, 6, 7, 8, 9, 10, 11, 12, 13, 17, 18, 19]. Significant variations in results following adult mouse ischemic injury often arise from discrepancies in experimental and statistical protocols. To address this, we developed a standardised strategy integrating Ki67, EdU, pH 3, and Aurora B to provide a comprehensive resolution of cardiomyocyte cell‐cycle progression. Within this framework, Ki67 serves as the primary indicator of initial cell‐cycle entry, while EdU and pH 3 mark S‐phase DNA synthesis and mitotic entry, respectively. Crucially, Aurora B kinase identifies cytokinesis at the midbody, which is essential for differentiating true cell division from polyploidization or abortive mitosis in the adult heart. While individual markers provide only transient snapshots, their integration facilitates tracking cardiomyocyte progression from initial activation to functional cytokinesis, establishing a robust framework for evaluating regenerative interventions. Notably, significant variations exist in the reported percentages of proliferating cardiomyocytes following regenerative therapies in adult mice with ischemic injury, primarily due to differences in experimental protocols and statistical methodologies. For example, the stimulated percentages of key proliferative markers after heart regeneration interventions are reported as follows: Ki67 positivity (0.08%–3.50%), EdU incorporation (0.16%–9.51%), pH 3 positivity (0.01%–0.64%), and Aurora B kinase expression (0.008%–1.75%) (Table 3). In comparison, the fold‐change differences in the percentages of proliferating cardiomyocytes relative to control groups show less variability: Ki67 positivity (3.5–12.5‐fold increases), EdU incorporation (2.2–5.6‐fold increases), pH 3 positivity (1.4–12.8‐fold increases), and Aurora B kinase expression (4.0–7.6‐fold increases) (Table 3). Therefore, while absolute counts per slide/area provide an alternative perspective [6, 12, 18, 19], we prioritise normalised percentages to account for variations in cell density and sample size across different tissue sections. This approach is supported by a high‐throughput framework that utilises whole‐slide scanning to analyse tens of thousands of cardiomyocytes, thereby eliminating the intra‐sample variability typical of traditional random‐field selection.

While multiparametric immunofluorescence provides necessary kinetic information, it remains a transient snapshot potentially confounded by cardiomyocyte polyploidization [29]. To overcome these inherent limitations, we implemented the MADM system as a permanent lineage‐tracing tool to provide definitive evidence of de novo cardiomyocyte production [7, 17, 30]. Unlike transient protein markers, the MADM system utilizes Cre‐mediated interchromosomal recombination during the G2 phase to distinctly label daughter cells with monochromatic green or red fluorescent reporters. In our framework, the appearance of monochromatic “twin spots” provides unambiguous evidence of completed cytokinesis, whereas yellow double‐positive cells identify cells that underwent DNA replication without division. By utilizing an inducible Myh6‐MerCreMer driver, this genetic labeling effectively captures the fate of dividing cells during specific post‐ischemic windows. Consequently, these methodologies offer a balanced workflow where multi‐parametric immunofluorescence serves as a cost‐effective primary screening tool, and the MADM system provides rigorous secondary validation to confirm that observed regenerative effects represent genuine hyperplasia rather than isolated nuclear events.

As a direct consequence of cardiomyocyte proliferation, the magnitude of increase in cardiomyocyte numbers serves as a critical indicator for assessing heart regeneration levels. This study details the efficient and standard enzymatic digestion methodology for isolating cardiomyocytes. In addition to this method, both Langendorff and non‐Langendorff perfusion techniques were utilised for cardiomyocyte isolation and subsequent quantification [21, 22]. Across various heart regeneration studies, hearts receiving regenerative interventions exhibited 1.15‐ to 1.88‐fold increases in cardiomyocyte numbers compared with controls [3, 5, 6, 17, 21, 30]. These findings underscore that successfully promoting endogenous cardiomyocyte proliferation ultimately manifests directly in an increase in cardiomyocyte numbers.

**FIGURE 3:**
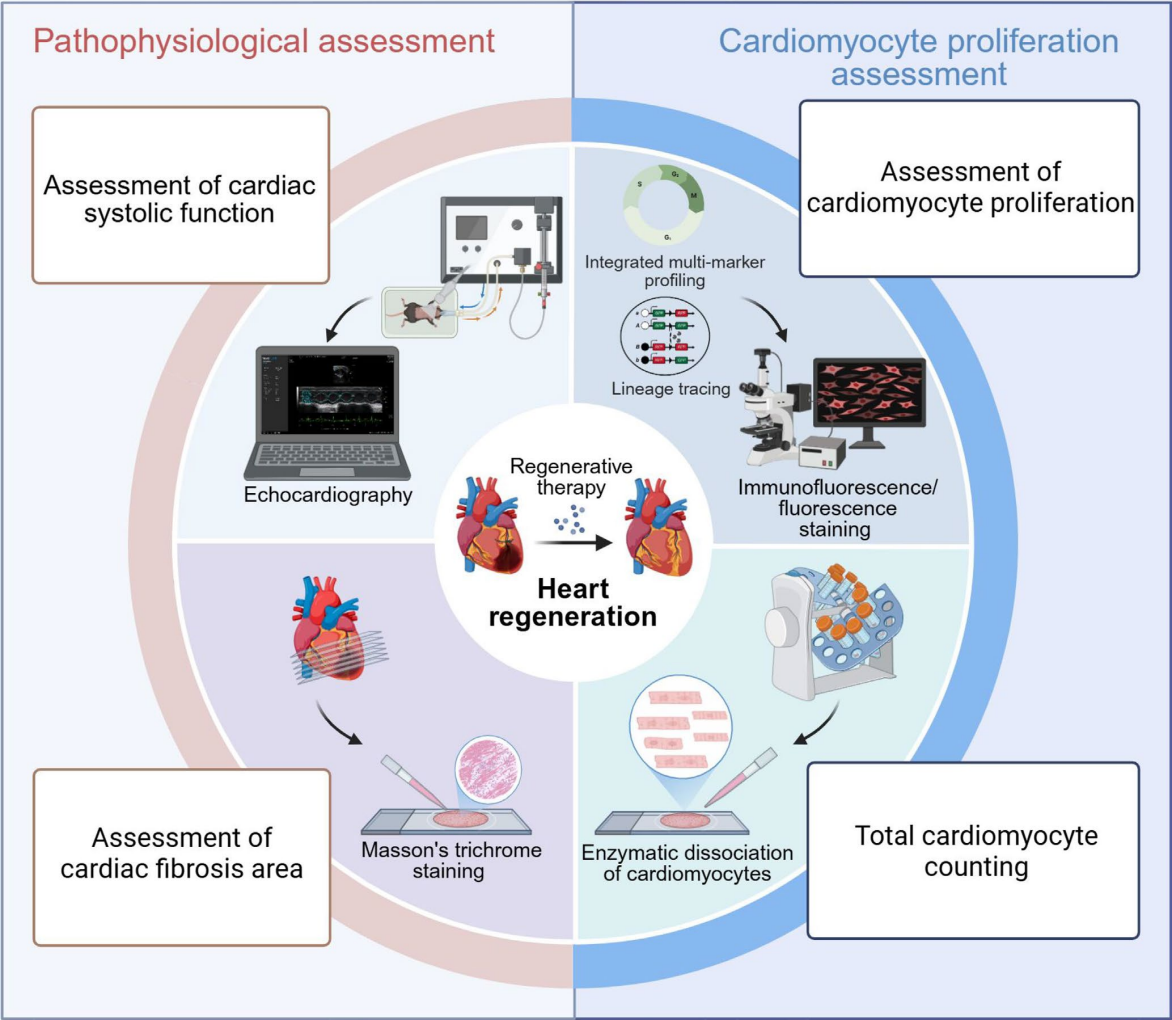
The integrated framework for assessing heart regeneration.

In conclusion, we established a standardised evaluation system to assess heart regeneration after ischemic injury in adult mice. The system integrates pathophysiological assessment with cardiomyocyte proliferation analysis, offering a multidimensional approach for quantifying regenerative therapeutic efficacy (Figure 3). This framework provides researchers with a unified methodology to accurately evaluate the effectiveness of heart regenerative therapies.

## Author Contributions

Y.L. and X.L. developed the methodology and drafted the manuscript. X.Z., Y.N., and J.F. supervised the project and revised the manuscript.

## Conflicts of Interest

The authors declare no conflicts of interest.

## Data Availability

Data sharing not applicable to this article as no datasets were generated or analysed during the current study.
